# Innovative Enterprises Development and Employees’ Knowledge Sharing Behavior in China: The Role of Leadership Style

**DOI:** 10.3389/fpsyg.2021.747873

**Published:** 2021-10-21

**Authors:** Daokui Jiang, Zhuo Chen

**Affiliations:** ^1^Business School, Shandong Normal University, Jinan, China; ^2^School of Innovation and Entrepreneurship, Shandong University, Jinan, China

**Keywords:** transformational leadership, authoritative leadership, explicit knowledge sharing, implicit knowledge sharing, organizational culture

## Abstract

Leadership is generally considered helpful for team knowledge sharing. However, differences in the influence of different leadership styles on team knowledge sharing mechanism is still unclear. To understand different leadership style foster team knowledge sharing, this study focuses on leader–follower trust during team interactions. From the perspective of leadership as social problem solving, we argue that transformational leadership and authoritative leadership are different linked to team knowledge sharing. Through the collection of a sample of 791 valid questionnaires in China, we used the structural equation model to test the theoretical model. Results showed that: (1) Transformational leadership was positively linked to explicit and implicit knowledge sharing, while authoritative leadership was positively linked to explicit knowledge sharing. (2) Trust tendency mediates the relationship between authoritative leadership and knowledge sharing. (3) Supportive and bureaucratic culture moderate the influence of trust tendency on implicit knowledge sharing, such that the positive relationship is stronger for the low-quality of supportive culture and the high-quality of bureaucratic culture. Finally, The study’s implication for theory and practice were discussed, its limitations were identified, and directions for future research were suggested.

## Introduction

As we all know, knowledge is an important means for organizations to acquire resources and maintain competitive advantages. Knowledge can be owned by an employee or a team. By building teams, knowledge sharing enables individuals to collaborate on expertise and skills that contribute to organizational value and competitive advantage. Teams can help organize values and competitive advantages (Alsharo et al., 2016). Research on knowledge management shows that employee knowledge sharing helps improve organizational performance. Social exchange theory believes that people behave in a way that maximizes their benefits and minimizes their costs. However, knowledge sharing depends on the willingness of team members to share their unique knowledge. Unfortunately, research generally suggests that sharing knowledge can lead to the loss of ownership of knowledge, which in turn can lead to loss of benefits. Team members refusing to share knowledge can hinder team cooperation, leading to failed goals. Therefore, the display of employees’ knowledge or ability is inseparable from good organizational culture and scientific leadership (Li et al., 2015). The research on leadership style explores the reasons why leaders are important to the organization. Leaders can influence the culture, atmosphere, vision and values of the organization through their own leadership behaviors, and develop a series of incentive systems (Zhu et al., 2011; Than et al., 2020). Leadership behavior also affects attitudes, trust, values (Le and Lei, 2017, 2018; Inceoglu et al., 2018), and ultimately affect knowledge sharing behavior (Bai et al., 2016; Le and Than, 2020).

Prior research studies highlighted different leadership approaches as an important factor for enhancing employee knowledge sharing behavior. Especially, the transformational leadership approach is considered the most suitable style to nurture followers’ attitudes in the context of organizational change. Previous studies have explored various factors influencing knowledge sharing behavior. However, previous studies did not distinguish the differences of knowledge sharing behaviors between different leadership styles. Knowledge sharing is divided into explicit and implicit knowledge sharing (Yong et al., 2013; Le and Lei, 2019; Nguyen et al., 2021). Explicit knowledge sharing relies more on the same language, rules of thumb, and conceptual framework, and can quickly spread its adopted methodology to all employees. Implicit knowledge sharing generally exists in highly professional fields, involving deep and almost intuitive understanding, which is difficult to express clearly. Explicit and implicit knowledge sharing are different in essence. Throughout the domestic and international research on the impact of leadership style on employee knowledge sharing behavior, it is rarely based on the Chinese sample. Previous research has emphasized the importance of organizational culture for organizational change. Organizational culture can motivate members of the organization to accept organizational values and promote organizational commitment to organizational commitment. Different from Western individualistic culture, Chinese mainstream culture is collectivism, and the influence of different leadership styles on employee behavior may be different.

The psychological contract is an unwritten agreement between the employee and employer, and psychological contract develops bonding between the leader and followers, which ultimately drives followers (who realize their leaders as trustworthy) performance like a champion in the context of organizational change (Gui et al., 2021; Lei et al., 2021). Given the important role of knowledge sharing in teamwork, we will use structural equation model to study the effect of leadership on team knowledge sharing behavior. We specifically explore the effect of different leaders on trust tendency and team knowledge sharing. Among them, there are two types of leadership styles, namely, transformational leadership and authoritative leadership. Correspondingly, there are two types of team knowledge sharing, namely implicit knowledge sharing and explicit knowledge sharing (Coun et al., 2019). We also explore the moderating effect of different organizational cultures on the above influencing processes. This study complements the leadership theory and knowledge management theory, and provides a reference for how to improve team knowledge sharing in different contexts. Organizations can take appropriate measures according to their own circumstances, in order to effectively encourage employees to share knowledge in the organization. Increasing the breadth of knowledge sharing among team members and increasing the depth of knowledge sharing among team members can help encourage and increase organizational competitive advantage.

The remainder of this paper proceeds as follows. The section following builds the theoretical model, employing prior literature on the sources of transformational leadership, authoritative leadership, types of organizational cultural, and knowledge sharing. Subsequently, the research methodology, data collection procedures, variables measurement and the respondent sample are described. Results of construct validation and model testing employing structural equation model are then reported. The paper concludes with a summary of study findings, highlighting contributions, implications, limitations and directions for future research.

## Literature Review and Hypotheses

### Leadership Style and Knowledge Sharing Behavior

In the era of knowledge economy, the acquisition, storage and utilization of knowledge resources have become the key factors for organizations to gain competitive advantage. Many organizations have established knowledge management systems to facilitate the flow of knowledge. But often it has little effect. The reason lies in the insufficient grasp of knowledge sharing by leaders, and the research on the relationship between transformational leadership and employee knowledge sharing is still insufficient.

Knowledge sharing behavior refers to the process in which knowledge is reproduced in its original or new form. That is to say, the individual or organization selectively transmits the knowledge that it possesses to other individuals or organizations in an appropriate manner. According to the classification of knowledge, knowledge includes two modes: explicit and implicit knowledge. Explicit knowledge is shared by meetings and documents. In contrast, implicit knowledge can be shared through interpersonal communication, interaction and cooperation. For organizations, the transfer of knowledge between knowledge providers and receivers not only simply completes the access and sharing of knowledge, but also transforms and increases the information and experience gained by both sides in the exchange and interaction. Implicit knowledge is highly personal knowledge, which is deeply rooted in the behavior itself and the environment of the individual. Implicit knowledge mainly includes individual thinking mode, belief proposition and mental mode (Nonaka, 1994). The exchange and sharing of implicit knowledge are closely related to the environment. Only when we are at the scene, on the spot, and on the interactive communication can we effectively transmit and share implicit knowledge.

Transformational leadership can better enable employees to realize the importance of their tasks and achieve results that exceed their original expectations. Transformational leaders know how to inspire the high-level needs of their subordinates, establish an atmosphere of mutual trust, and encourage subordinates to sacrifice their own interests for the benefit of the organization (Longshore and Bass, 1985; Burns et al., 1999; Galante and Ward, 2017; Oluwatosin and Olumide, 2017; Spies et al., 2018). Transformational leadership is conducive to improving the work efficiency of employees (Boamah et al., 2017), stimulating their employment opportunities (Wang et al., 2017), promoting the promotion of employees’ positions (Hetland et al., 2018), and reducing their intention to leave (Eberly et al., 2017). Clearly, transformational leadership has a positive impact on the organization (Elrehail et al., 2018; Ojha et al., 2018).

Transformational leadership is mainly manifested in two aspects. On the one hand, it creates a personalized environment for employees by influencing and changing organizational culture. On the other hand, through effective communication and empowerment between leaders and employees through their own charm, employees’ enthusiasm can be enhanced to stimulate their potential, thus forming a positive interaction between leaders and subordinates (Niessen et al., 2017). Leadership, as the core of a team, has an important impact on employee knowledge sharing. Previous studies have shown that transformational leadership can effectively promote knowledge sharing. First, transformational leaders establish moral paradigms within organizations through behavioral demonstrations. Under the role of moral paradigm, it is easy for members to form an atmosphere of mutual trust. Employees are willing to follow and emulate the high ethical standards of transformational leadership and tend to share their knowledge and skills with other members. Secondly, leaders know how to stimulate employees with incentives better, so that employees remain optimistic and confident. At the same time, transformational leadership also encourages employees to sacrifice their own interests for the sake of organizational interests, so that employees tend to share their knowledge to serve the organization on the premise of achieving their own goals. Third, transformational leadership encourages innovation, nurturing, and developing independent thinking employees. It is also very popular for employees to actively publish, share their ideas, and innovate knowledge. Fourthly, the personalized care behavior of transformational leadership promotes the intention of sharing knowledge sharing. Because of the open communication between transformational leaders and employees, and the emphasis on employees’ opinions and needs, employees have a strong sense of belonging and trust in the organization.

Transformational leaders show more private care to their subordinates. Transformational leadership creates a friendly atmosphere of mutual help and mutual promotion in a team. Compared with traditional leaders, transformational leaders show more personal care to subordinates, which can better make employees aware of the importance of work and stimulate higher-level demands of subordinates. Working with transformational leaders, employees are willing to put in extra effort for the job (Purvanova and Bono, 2009; Lehmann-Willenbrock et al., 2015; Aga et al., 2016). The positive and spontaneous team atmosphere created by transformational leaders provides a trust and trustworthy environment for knowledge sharing, which greatly promotes knowledge sharing.

*Hypothesis 1a*: Transformational leadership has significant effects on explicit and implicit knowledge sharing behavior.

The authoritative leader, the leader, requires his subordinate employees to obey themselves absolutely, emphasizing that they have absolute control power and decision-making power. If authoritative leadership is adopted in an organization, subordinates usually respond directly to obedience, awe and distance (Nicol, 2009). Authoritative leadership has four notable characteristics. (1) The authoritarian style is the authoritative leadership dictatorship, and the control is completely in their hands. (2) Degrading subordinate abilities is an active behavior for subordinate employees. Authoritative leaders often ignore them and emphasize their authority. (3) Image consolidation means that authoritative leaders attach importance to their image in subordinates, and show an image conducive to the establishment of their own authority. (4) Educational behavior, that is, authoritative leadership often reprimands employees, and criticizes employees when problems arise rather than reflecting on themselves. Correspondingly, subordinates will respond to the behavior of authoritative leaders by obeying obediently, respecting and fearing, unconditionally obeying and shaming.

In the Chinese context, the distribution of authoritative leadership and emotional care have an impact on employees’ willingness to share (Chen et al., 2018). When leaders adopt authoritative leadership behavior, some behaviors show an autocratic and controlling side, which is not conducive to knowledge sharing behavior of employees. Another aspect of authoritative leadership lies in the fact that leadership is a reward and punishment. The authoritative leadership will formulate corresponding incentive and punishment systems, which are authoritative for employees, and employees will also show compliance, awe, and change. Therefore, if an authoritative leader attaches importance to knowledge management in the organization and promotes a culture of knowledge sharing, employees will respond positively because of the fear of others. From this perspective, authoritative leaders can also have a positive impact on knowledge sharing behavior.

The stickiness of implicit knowledge makes it take more time and energy to share implicit knowledge than explicit knowledge. The transformation from implicit knowledge to explicit knowledge is a typical innovation process. This process is accompanied by the transformation of experience, intuition and imagination into language, as well as the transformation of perceptual knowledge into rational knowledge, as well as the creation of new knowledge and the invention of new technology. In this process, the implicit knowledge of the individual is transformed into explicit knowledge, which can be referenced and shared by other members. Moreover, these explicit knowledge can be subtly entered into the implicit knowledge of the individual through learning and understanding, thus forming the transformation within the individual. The authoritarian style of authoritative leadership, depreciation of subordinates’ abilities and image rectification will make subordinates feel authoritarian and arbitrary. As a result, in terms of knowledge sharing, employees in an organization may be dissatisfied with the autocratic leadership and unwilling to contribute their knowledge to the organization. But there are also instigations in authoritative leadership. Authoritative leaders require high performance of leaders and blame for low-performance behaviors, and some guidance behaviors provided to employees, which also gives employees a sense of reverence and thus obeys the requirements of leaders. Therefore, when the authoritative leadership in the organization promotes knowledge sharing, the team members will perform as required due to the awe of the leader.

*Hypothesis 1b*: Authoritative leadership has significant effects on explicit and implicit knowledge sharing behavior.

### The Mediating Role of Trust Tendency

Trust is described as “the tendency to be willing to risk other groups or individuals when faced with uncertainties” (Kong, 2017; Burt et al., 2018). Trust is especially important for the achievement of organizational goals when many people work together to accomplish their work (Allen et al., 2018). Trust is an important informal governance mechanism in the relationship between individuals within an organization (Griessmair et al., 2014; Justwan et al., 2017). Trust is an important part of human social life (Haas et al., 2015).

The trust tendency among members helps create a positive organizational climate. This atmosphere may affect employee’s work attitude, such as employee satisfaction and organizational commitment (Miao et al., 2016). As trust increases, employees and managers work together, which is more conducive to making informed choices and ultimately achieving job performance (Janssen and Yperen, 2004). When the level of trust in the organization is high, the employee’s ability can be well developed, and the organizational goals and personal goals can be easily achieved. On the contrary, in the absence of confidence, employees are unwilling to engage in positive work behavior, which is not conducive to the achievement of job performance. Generally speaking, people in a highly trusted environment are more willing to participate in knowledge sharing and social exchange. Trust relationship is the key factor that affects team members’ knowledge sharing. Trust relationship can directly affect team members’ knowledge sharing, or indirectly affect knowledge sharing by influencing other factors (Yao et al., 2017).

Many scholars have shown through empirical evidence that when social relations between people are highly trusted, they are generally more willing to participate in social interaction and knowledge sharing. Interpersonal trust has a positive effect on the knowledge sharing of team members (Chang and Chuang, 2011; Tamjidyamcholo et al., 2014). As one of the factors affecting user knowledge sharing, trust has a positive impact on knowledge sharing behavior. Trust is a state of mind. Knowledge sharing can be seen as a cooperative behavior. Because of the existence of trust, team members are more likely to have a cooperative relationship. According to social cognition, individual behavior, subject cognition and environment are determined by dynamic interaction. That is, trust can directly affect people’s knowledge sharing behavior, and can also have an indirect effect on user behavior through subjective cognition. Among them, subject cognition includes perception of self behavior and team perception. Team members’ perception of knowledge sharing behavior includes knowledge sharing self-efficacy, perceived relative and perceived compatibility. Trust can also indirectly influence team members’ knowledge sharing through team perception. Employees’ perception of the team includes their sense of belonging and attachment. Positive cognitive trust and emotional trust encourage members to participate in team work spontaneously and do their best for the survival and development of the team without any reward.

*Hypothesis 2a*: Trust tendency mediates the effects of transformational leadership on explicit and implicit knowledge sharing behavior.

*Hypothesis 2b*: Trust tendency mediates the effects of authoritative leadership on explicit and implicit knowledge sharing behavior.

### The Moderating Role of Organizational Culture

Organizational culture refers to the comprehensive system of values, beliefs, norms, basic assumptions and behaviors shared by members of the organization. The value of the organization plays an important role in guiding the behavior of the members of the organization and is an important basis for the formation of behavioral norms. Because each organization’s structure is different from its environment, different organizational culture types are created. From the perspective of communication and interaction among the members, organizational culture can be divided into innovative culture, supportive culture and bureaucratic culture (Wallach, 1983). Nowadays, with the increasingly fierce competition outside the organization, more and more organizations are inclined to establish a high-performance organizational culture (Shoham et al., 2012; Ali and Park, 2016; Matinaro and Liu, 2017). Organizational culture is the deep reflection of the values and beliefs of the organization members. Organizational culture profoundly affects the preferences of individual knowledge choice and the way of knowledge acquisition. On this basis, organizational culture further influences the creation, transfer and sharing of knowledge.

Innovative culture means that the main belief of an organization is innovation. Innovative culture is a combination of various cultural forms related to innovation practice, with the pursuit of change, advocating innovation as the basic concept and value orientation (Cameron and Quinn, 1999; Tripathi and Tripathi, 2009). With the rapid shortening of the half-life of the advantages of products and services, the ability to actively change and innovate becomes one of the key factors for the success of the team and organization. More and more researches emphasize that organizational culture is the key to management innovation (Khazanchi et al., 2007; Škerlavaj et al., 2010). Organizations can strive to establish a team knowledge sharing mechanism with project or goal as the core through innovative culture. This mechanism provides everyone with the opportunity to participate in the creation process of the organization’s proprietary knowledge, and also creates more opportunities for group production and invention, enabling more people to share team knowledge. This method of constructing innovative culture not only helps to cultivate talents and disseminate innovative ideas, but also avoids the loss caused by the monopoly of the proprietary knowledge of a minority organization.

Innovative culture has the following characteristics: encouraging the exchange of experience between people, emphasizing the spirit of teamwork, paying attention to the needs of employees. In this atmosphere, knowledge sharing is naturally carried out among employees. When an individual’s social network forms an innovative culture, the individual team is encouraged to face up to challenges and innovations. In such a group, individuals can be more tolerant of failure and respect for differences (Li et al., 2014). Organizational innovation is mainly driven by the interaction and transformation between implicit knowledge and explicit knowledge. In particular, the flow and sharing of implicit knowledge is the key to organizational innovation. The acquisition of implicit knowledge is an immersive experience, a process of understanding and self-correction, and a process of enriching and perfecting implicit knowledge. The scope of implicit knowledge is restricted because of its inexpressible characteristics. It is necessary for organizations to take measures to promote the flow and sharing of implicit knowledge. More and more studies emphasize that organizational culture is the key to management innovation.

*Hypothesis 3a*: Innovative culture moderates the effects of trust tendency on explicit and implicit knowledge sharing behavior, such that the effects of trust tendency on explicit and implicit knowledge sharing behavior are stronger (vs. weaker) with stronger (vs. weaker) innovative culture.

Supportive culture emphasizes participation, collaboration and people-oriented. Focusing on group cohesion and personal growth, encouraging employees to express their views on work and others, and attaching importance to each employee’s identification with the organization are all characteristics of a supportive culture. A supportive culture can create a sense of family-like warmth for the organization. Why do we say that? Because in an organization with a supportive culture, organizations and leaders have high support and trust for employees. Not only that, organizations and leaders also value employee engagement, teamwork and interpersonal relationships. There is a spirit of mutual cooperation and cooperation among members. “Knowledge sharing” is a more appropriate expression than knowledge transfer. It is used to describe knowledge sharing and emphasize knowledge sharing within groups and teams (Ryan and O’Connor, 2013). Social interaction among team members is regarded as a means to acquire and share knowledge. Organizational culture, especially organizational values, has great influence on employees’ attitudes and behaviors. In organizations that use frankness, openness, and communication as the underlying principles, employees trust and intimate each other and are passionate about knowledge sharing. After a large amount of knowledge is internalized into the organization, the role is amplified to stimulate and assist innovation. A supportive cultural team will form an effective guarantee mechanism to ensure a highly supportive, fair and harmonious working environment within the organization. This kind of work environment will promote full communication between the teams. There are many things that organizations can do to make it easier for employees to generate knowledge-sharing behaviors. For example, organizations can support and create a trusted environment, use open communication methods and empowerment to employees, and establish a culture of cooperation and mutual learning.

*Hypothesis 3b*: Supportive culture moderates the effects of trust tendency on explicit and implicit knowledge sharing behavior, such that the effects of trust tendency on explicit and implicit knowledge sharing behavior are stronger (vs. weaker) with stronger (vs. weaker) supportive culture.

Bureaucratic culture is usually a hierarchical organization. Bureaucratic culture is characterized by clear hierarchy, clear responsibility and authorization, systematic work and immobilization. This kind of culture is based on control and power, and the organization is often stable. In general, organizations emphasize more on following rules than changing rules. The organization requires the staff to carry out the work strictly according to the business process set by the company and conduct examination. If employees fail to perform their duties according to the company’s standards, they may be punished. Bureaucratic culture is characterized by responsibility and power. The management of bureaucratic cultural organizations is more based on organizational control and power division. In bureaucratic culture organizations, the flow of information and power is based on system and hierarchy (Meijer, 2008; Eshbaugh-Soha, 2017; Filho and Waterson, 2018). The bureaucracy of a bureaucratic culture is characterized by cumbersome organization and poor information exchange, which can hinder the team’s knowledge sharing. The higher the degree of formalization, complexity or centralization of the organization, the more unfavorable the occurrence of knowledge sharing behavior. The organizational structure of bureaucratic culture is hierarchical, with clear division of functions and responsibilities, and the work nature is mainly standardized and fixed. The motivation of knowledge sharing cannot be demonstrated in the process of bureaucratic organization operation. The culture of attaching importance to records (bureaucratic and hierarchical culture) can effectively store employees’ explicit knowledge, but can not effectively achieve implicit knowledge sharing. Excessive institutional constraints and overly complex organizational structures can have a negative impact on knowledge sharing across departments or individuals within an organization. Organizational culture influences and regulates the values and behaviors of employees. In turn, employees’ attitudes and behaviors toward knowledge sharing are bound to be influenced by organizational culture. Therefore, it is of great practical significance to seek and establish an organizational atmosphere and rules suitable for knowledge sharing.

*Hypothesis 3c*: Bureaucratic culture moderates the effects of trust tendency on explicit and implicit knowledge sharing behavior, such that the effects of trust tendency on explicit and implicit knowledge sharing behavior are stronger (vs. weaker) with stronger (vs. weaker) bureaucratic culture.

**FIGURE 1:**
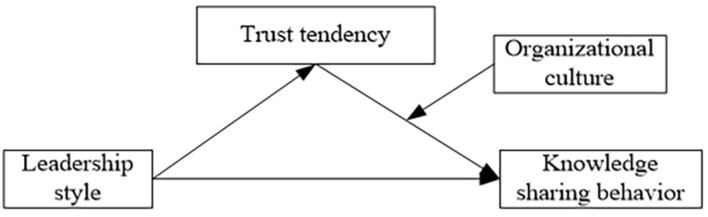
Research model.

The research model of this paper is shown in Figure 1.

## Materials and Methods

### Participants and Procedure

Data collection for the main study was conducted by online. After the questionnaire was designed on the star platform, we sent links to friends and colleagues and explained our subjects to them, asking them to help send questionnaires to people working in different industries. Each participant was assigned an anonymous randomized code to ensure privacy and increase response rate. And a geographic IP restriction was used to ensure no one could access the questionnaire twice. Finally, a total of 791 valid questionnaires (team work experience) were collected by setting items and eliminating unreasonable questionnaires. All assessments were conducted in Chinese.

### Measures

We used scales established by previous researchers for measures. Model constructs were rated on a five-point Likert scale ranging from 1 (strongly disagree) to 5 (strongly agree). At the same time, we examined gender, age, educational level and years working in colleges as controlled variables. Transformational leadership was measured using the 4-item scale (Podsakoff et al., 1990). The Cronbach’ ɑ of this scale was 0.851. Authoritative leadership was measured using the 4-item scale from Chou et al. (2005). The Cronbach’ ɑ of this scale was 0.839. Innovative culture, supportive culture and bureaucratic culture were measured using the 4-item scale from Quinn and Rohrbaugh (1983) and Claver et al. (1999). The Cronbach’ ɑ of this scale were 0.840, 0.880, and 0.869. Trust tendency was measured using a 4-item scale adapted from Singh and Srivastava (2009). The Cronbach’ ɑ of this scale was 0.876. Explicit and implicit knowledge sharing were measured using the 4-item scale from Bock et al. (2005). The Cronbach’ ɑ of this scale were 0.875 and 0.882.

### Descriptive Statistical Analysis

The demographic variables were described and analyzed. The final data set had 160 valid responses. This sample comprised 433 (54.7%) males and 358(45.3%) females. In terms of age, 11.8% (93 persons) interviewed under 25 years old, 33.1% (262 persons) aged 26–30 years old, 43.7% (346 persons) aged 31–40 years old, 11.4% (90 persons) over 41 years old. In terms of academic qualifications, 66.1% (523 persons) have undergraduate degree, followed by 25.4% (201 persons) with junior college degree or below, and 8.5% (67 persons) have the graduate degree. In terms of working hours, 8.1% (64 persons) worked under 1 year, 32.5% (257 persons) for 1–5 years, 21.9% (173 persons) for 5–10 years and 37.5% (297 persons) for more than 10 years. In terms of the team type, 24.0% (190 persons) are in the product development team, 38.2% (302 persons) in the market development team, and 37.8% (299 persons) in the engineering and technology team.

## Results

### Reliability and Validity Analysis

We mainly test internal consistency reliability, composite reliability, convergent validity and discriminant validity. Internal consistency reliability is measured by the Cronbach ɑ. Composite reliability is measured by the CR value. Convergent validity is measured by average variance extracted (AVE) values. Discriminant validity is measured by the difference between the square root of AVE and the correlation coefficient.

The results show that the *Cronbach ɑ* coefficient is greater than 0.6, and the *CR* is greater than 0.6, and the *AVE* is greater than the acceptance threshold of 0.36–0.5 (Fornell and Larcker, 1981). At the same time, the square root of the AVE value is greater than the correlation coefficient value of the row and column. Based on the above judgment, the reliability and validity of the scale meet the requirements. The numerical value of the diagonal is the AVE of the factor construct, and the numerical value of the lower triangle is the correlation coefficients between constructs. The reliability, validity and correlation coefficient matrix of the variables are shown in Tables 1, 2.

**TABLE 1:** Correlation coefficient matrix of the variables and discriminant validity.

	GEN	AG	LE	DE	TML	ATL	INC	SPC	BUC	TRU	EXK	IMK
GEN	1											
AG	−0.21**	1										
LE	−0.24**	0.79**	1									
DE	0.06	–0.03	–0.06	1								
TML	0.10**	0.08*	0.10**	0.03	(0.82)							
ATL	0.09*	0.021	0.03	0.06	0.58**	(075)						
INC	0.06	0.04	0.06	0.05	0.52**	0.55**	(075)					
SPC	0.13**	0.04	0.05	0.07	0.48**	0.56**	0.66**	(081)				
BUC	0.07*	0.02	0.05	0.03	0.45**	0.52**	0.62**	0.64**	(079)			
TRU	0.05	0.06	0.04	0.08*	0.41**	0.52**	0.52**	0.56**	0.52**	(080)		
EXK	0.09*	0.01	0.01	0.07	0.44**	0.50**	0.53**	0.55**	0.52**	0.59**	(080)	
IMK	0.08*	0.04	0.07*	0.03	0.44**	0.48**	0.55**	0.56**	0.53**	0.58**	0.66**	*(0.81)*

The test is a Pearson correlation two-tailed test. *p-value < 0.05; **p-value < 0.01; *** *p*-value < 0.001. Gen, gender; AG, age; LE, length of employ; DE, degree of education; TML, transformational leadership; ATL, authoritative leadership; INC, innovative culture; SPC, supportive culture; BUC, bureaucratic culture; TRU, trust tendency; EXK, explicit knowledge sharing; IMK, implicit knowledge sharing. Italics in parentheses are the square root of AVE.

**TABLE 2:** The composition reliability and convergent validity.

Variables	Items	Estimate				Square multiple correlations	Composition reliability	Convergent validity
		Std.	SE	*Z*-value	*P*-value	SMC	CR	AVE
TML	0.75	0.64	0.03	22.88	***	0.56	0.86	0.67
	0.89	0.76	0.03	28.61	***	0.79		
	0.80	0.70	0.03	24.85	***	0.64		
ATL	0.78	0.67	0.03	23.75	***	0.61	0.84	0.57
	0.78	0.66	0.03	23.76	***	0.61		
	0.71	0.61	0.03	20.79	***	0.50		
	0.74	0.66	0.03	22.31	***	0.55		
INC	0.70	0.61	0.03	20.36	***	0.49	0.84	0.57
	0.84	0.70	0.03	26.31	***	0.71		
	0.76	0.66	0.03	23.31	***	0.58		
	0.71	0.59	0.03	20.78	***	0.50		
SPC	0.83	0.74	0.03	27.02	***	0.69	0.88	0.65
	0.84	0.74	0.03	27.23	***	0.71		
	0.76	0.68	0.03	23.89	***	0.58		
	0.79	0.72	0.03	25.12	***	0.62		
BUC	0.81	0.70	0.03	26.02	***	0.66	0.87	0.63
	0.85	0.77	0.03	27.77	***	0.72		
	0.75	0.69	0.03	23.50	***	0.56		
	0.75	0.65	0.03	23.46	***	0.56		
TRU	0.82	0.73	0.03	26.20	***	0.67	0.88	0.64
	0.86	0.78	0.03	28.50	***	0.74		
	0.77	0.71	0.03	24.35	***	0.59		
	0.75	0.65	0.03	23.45	***	0.56		
EXK	0.80	0.70	0.03	25.59	***	0.64	0.88	0.64
	0.87	0.74	0.03	28.77	***	0.76		
	0.77	0.67	0.03	24.51	***	0.59		
	0.75	0.66	0.03	23.05	***	0.56		
IMK	0.86	0.72	0.03	28.58	***	0.74	0.89	0.66
	0.83	0.73	0.03	27.13	***	0.69		
	0.78	0.70	0.03	24.76	***	0.61		
	0.77	0.65	0.03	24.19	***	0.59		

***p-value < 0.001.

### Testing the Measurement Models

We use AMOS22.0 to verify discriminant validity among variables in confirmatory factor analysis. The scales used in this study are all based on the maturity scale developed by the predecessors, and have been empirically tested by scholars. By comparing the suitability of each model, it was found that the fit of the eight-factor model (χ^2^ = 1490.97, df = 406, χ^2^/df = 3.67, CFI = 0.94, IFI = 0.94, NFI = 0.92, TLI = 0.93, RMSEA = 0.06), was superior to other models (as shown in Table 3). Therefore, the eight-factor model was chosen as the basis for the analysis of this study.

**TABLE 3:** The measurement models analysis results.

Model fit	Criteria	One factor	Three factors	Five factors	Six factors	Seven factors	Eight factors
CMIN	—	2936.42	2233.21	1726.93	1648.24	1589.58	1490.97
DF	—	434	431	424	419	413	406
CMIN/DF	1<NC<5	6.77	5.18	4.07	3.93	3.85	3.67
CFI	>0.9	0.87	0.90	0.93	0.93	0.94	0.94
IFI	>0.9	0.87	0.90	0.93	0.93	0.94	0.94
NFI	>0.9	0.85	0.88	0.91	0.91	0.92	0.92
TLI	>0.9	0.85	0.89	0.92	0.92	0.92	0.93
RMSEA	<0.08	0.09	0.07	0.06	0.06	0.06	0.06

### Testing the Structural Models

We use AMOS22.0 to test hypotheses and construct structural equation model. Furthermore, we analyze the model by referring to the mediation analysis and moderated mediating analysis proposed by Preacher and Hayes (2004) and Zhao et al. (2010). This model demonstrated good fit with the data (χ^2^ = 811.21, df = 164, χ^2^/df = 4.94, CFI = 0.94, IFI = 0.94, NFI = 0.93, TLI = 0.93, RMSEA = 0.07).

As shown in Table 4, the results show that transformational leadership has a significant effect on explicit knowledge sharing behavior (β = 0.064, *p* < 0.05). Transformational leadership has a significant effect on implicit knowledge sharing behavior (β = 0.102, *p* < 0.000). Hypothesis 1a was supported.

**TABLE 4:** Structural equation analysis results.

D.V		ID.V	Estimate	SE	C.R.	*P-value*	Hypothesis
EXK	←	TML	0.064	0.032	1.975	0.048	1a ✓
IMK	←	TML	0.102	0.032	3.135	0.002	1a ✓
EXK	←	ATL	0.132	0.039	3.381	***	1b ✓
IMK	←	ATL	0.043	0.039	1.096	0.273	1b ×
TRU	←	TML	0.041	0.036	1.134	0.257	2a ×
TRU	←	ATL	0.274	0.041	6.631	***	2b ✓
EXK	←	TRU	0.412	0.032	12.879	***	2b ✓
IMK	←	TRU	0.457	0.032	14.223	***	2b ✓
TRU	←	INC	0.154	0.046	3.381	***	
TRU	←	SPC	0.26	0.042	6.125	***	
TRU	←	BUC	0.194	0.041	4.778	***	
EXK	←	INC	0.061	0.154	0.396	0.692	
IMK	←	INC	0.346	0.154	2.241	0.025	
EXK	←	SPC	0.436	0.143	3.047	0.002	
IMK	←	SPC	0.538	0.144	3.744	***	
EXK	←	BUC	–0.015	0.158	–0.095	0.924	
IMK	←	BUC	–0.272	0.159	–1.709	0.087	
EXK	←	TRUINC	0.211	0.04	0.773	0.439	3a ×
IMK	←	TRUINC	–0.294	0.04	–1.088	0.277	3a ×
EXK	←	TRUSPC	–0.616	0.039	–2.259	0.024	3b ✓
IMK	←	TRUSPC	–0.745	0.039	–2.762	0.006	3b ✓
EXK	←	TRUBUC	0.239	0.041	0.828	0.408	3c ×
IMK	←	TRUBUC	0.772	0.042	2.708	0.007	3c ✓

***p-value < 0.001.

Authoritative leadership has a significant effect on explicit knowledge sharing behavior (β = 0.132, *p* < 0.000). The effect of authoritative leaders on implicit knowledge sharing behavior is not significant (β = 0.043, *p* > 0.05). Hypothesis 1b was partially supported.

The effect of transformational leadership on trust tendency is not significant (β = 0.041, *p* > 0.05). Authoritative leadership has a significant effect on trust tendency (β = 0.274, *p* < 0.000). Meanwhile, the effect of trust tendency on explicit knowledge sharing (β = 0.412, *p* < 0.000) and implicit knowledge sharing (β = 0.457, *p* < 0.000) is significant. Based on the hypothesis of transformational leadership and authoritative leadership, we can draw the following conclusions. Trust tendency has no mediating effect on the influence of transformational leadership on explicit knowledge sharing and implicit knowledge sharing. Trust tendency has mediating effect on the influence of authoritative leadership on explicit knowledge sharing and implicit knowledge sharing.

Sobel test was used to test the existence of mediation effect. If Sobel is greater than 2 or less than −2, the mediation effect is significant. From Table 5, we can see that the mediating effect of trust tendency is not significant in the influence of transformational leadership on explicit and implicit knowledge sharing. Hypothesis 2a was not supported. Trust tendency is significant in the influence of authoritative leadership on explicit and implicit knowledge sharing behavior. Hypothesis 2b was supported.

**TABLE 5:** Mediating effect test.

Relationship of constructs	Estimate	SE	Sobel test	*P*-value
TML→TRU	0.041	0.036	1.134	0.257
TRU→EXK	0.396	0.032		
TML→TRU	0.041	0.036	1.135	0.256
TRU→IMK	0.449	0.031		
ATL→TRU	0.294	0.037	6.686	0.000
TRU→EXK	0.396	0.032		
ATL→TRU	0.294	0.037	6.966	0.000
TRU→IMK	0.449	0.031		

As shown in Table 4, the moderating effect of innovation-oriented culture on explicit knowledge sharing behavior (β = 0.211, *p* > 0.05) and implicit knowledge sharing behavior (β = −0.294, *p* > 0.05) is not significant, that is, hypothesis 3a was not supported. The moderating effect of supportive culture on the influence of trust tendency on explicit knowledge sharing behavior (β = −0.616, *p* < 0.05) and implicit knowledge sharing behavior (β = −0.745, *p* < 0.01) is not significant. Hypothesis 3b was supported. The moderating effect of bureaucratic culture on the influence of trust tendency on dominant knowledge sharing behavior (β = 0.239, *p* > 0.05) is not significant, and it is significant in the influence of trust tendency on implicit knowledge sharing behavior (β = 0.772, *p* < 0.01). Hypothesis 3c was partially supported.

**FIGURE 2:**
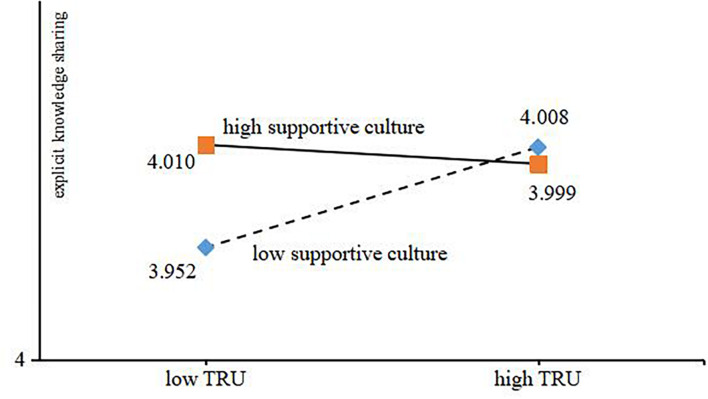
Moderating effect of supportive culture.

**FIGURE 3:**
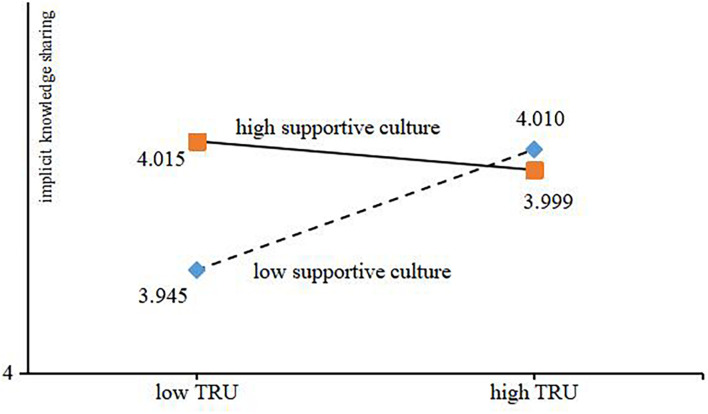
Moderating effect of supportive culture.

In order to describe the moderating role of supporting culture in the process of the influence of trust tendency on explicit and implicit knowledge sharing, Figures 2, 3 depict this influence relationship. The results showed that under the background of high supportive culture, the higher the level of trust tendency, the less the explicit and implicit knowledge sharing behavior. Under the background of low supportive culture, the higher the level of trust tendency, the more explicit and implicit knowledge sharing behaviors.

**FIGURE 4:**
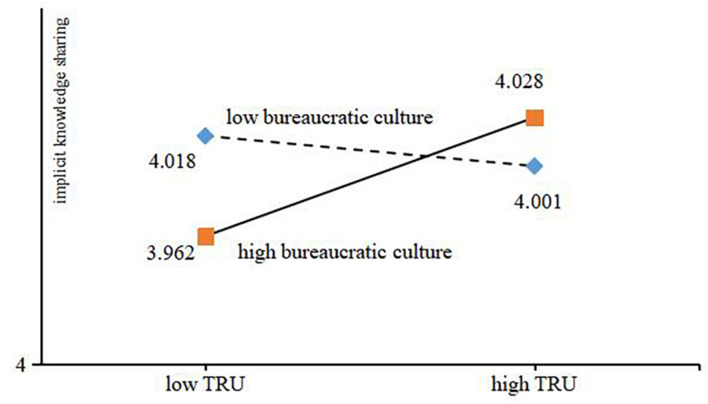
Moderating effect of bureaucratic culture.

At the same time, in order to describe the moderating role of bureaucratic culture in the process of trust tendency to implicit knowledge sharing, Figure 4 depicts this influence relationship. The results show that in the context of high bureaucratic culture, the higher the level of trust tendency, the more implicit knowledge sharing behavior. Correspondingly, in the context of low bureaucratic culture, the higher the level of trust tendency, the less implicit knowledge sharing behavior.

## Conclusion

Organizations are increasingly leveraging the skills and knowledge of their employees in the form of teams. However, the research on how different leadership styles affect knowledge sharing behavior is insufficient. This study proposes a conceptual model of the relationship among transformational leadership, authoritative leadership, trust tendency, explicit and implicit knowledge sharing. Based on social exchange theory, this paper establishes a theoretical model. The results show that transformational leadership has a significant effect on explicit and implicit knowledge sharing behavior. Correspondingly, authoritative leadership has a significant impact on explicit knowledge sharing behavior, while authoritative leadership has no significant impact on implicit knowledge sharing behavior. Sobel test showed that the influence of trust tendency mediated authoritative leadership on explicit knowledge sharing behavior. Contrary to expectations, trust tendency mediate the influence of transformational leadership on knowledge sharing behavior is not significant. Trust is still a complex phenomenon, and the team’s characteristics add to that complexity. Knowledge sharing behavior in team environment is an important factor for team members to establish social capital and social exchange. A team is considered to be a place where members share knowledge and change the environment to gain new insights. This study expands the literature on team theory, leadership theory, and knowledge management theory, and believes that leadership is critical to team members’ knowledge sharing and trust relationships.

This study also explores the influence of trust tendency on explicit and implicit knowledge sharing in the context of innovative culture, supportive culture and bureaucratic culture. The results show that the supportive culture moderates the effect of trust tendency on explicit and implicit knowledge sharing behavior, and the bureaucratic culture moderates the effect of trust tendency on implicit knowledge sharing behavior. Contrary to expectations, the innovative culture has no significant moderating effect on the relationship between trust tendency and knowledge sharing behavior.

## Discussion

### Theoretical Implications

First, quite a few previous studies have highlighted the key role of leadership in knowledge sharing, but most knowledge-sharing studies focus on Western leadership styles while ignoring non-Western perspectives. However, non-Western and Western leadership styles are very different, and their responses to employees have their own unique influences. Incorporating non-Western leadership style into the knowledge sharing theory model is conducive to the integrity of knowledge sharing research. In this study, authoritative leadership in China is a common leadership style in organizations. As a prerequisite for knowledge sharing, it explains the mechanism of authoritative leadership’s influence on knowledge sharing behavior.

Secondly, this study proposes a new process model. Transformational leadership has a direct impact on team knowledge sharing behavior, while authoritative leadership promotes knowledge sharing behavior by improving the level of team trust tendency. Previous empirical studies have shown that transformational leadership has a direct impact on explicit and implicit knowledge sharing behavior. This study also examined the impact of authoritative leadership on explicit and implicit knowledge sharing behavior. In the context of Chinese leadership style, authoritative leadership influences implicit knowledge sharing behavior by enhancing team trust. In the Chinese leadership style, in order to promote the implicit knowledge sharing behavior of employees, it is necessary to focus on the factors that influence the team’s trust tendency, especially the leadership factors.

Thirdly, supportive culture and bureaucratic culture moderate the influence of trust tendency on knowledge sharing behavior, while innovative culture has no such moderating effect. In the context of Chinese culture, relationship orientation is dominant. In the case of fixed trust level, innovative culture has no significant influence on knowledge sharing behavior. In other words, the choice of knowledge sharing has nothing to do with the innovation culture, but is directly influenced by the level of trust. However, in the context of supportive culture and bureaucratic culture, the influence of trust on knowledge sharing behavior is significantly different.

### Practical Implications

First, consistent with other relevant research findings, transformational leadership has a significant impact on employee creativity, adaptability, and employee motivation. That is, this study shows that transformational leadership has a significant impact on promoting team knowledge sharing behavior. Transformational leadership can increase employee knowledge, skills, employee attitudes, and other employee and team behaviors that contribute to job performance. Organizations can create a good environment, such as an effective system, and leaders can show more transformational behavior, such as communicating a convincing vision and encouraging employees to participate in management. Authoritative leadership is a double-edged sword, which is effective in specific environment, but its influence on explicit and implicit knowledge sharing behavior is different. When managers show authoritative leadership style, team members show explicit knowledge sharing behavior, but only in the promotion of trust level, team members show implicit knowledge sharing behavior.

Secondly, trust is crucial to organizational development. Afsar et al. (2015) explores the relationship between trust factors and environmental matching, employee innovative work behavior, and job performance. The results show that people-post matching and People-Organization matching have a positive impact on innovation behavior through innovation trust, and ultimately promote innovation performance, in which innovation trust plays a mediating role. This study found that the influence of trust tendency mediated authoritative leadership on implicit knowledge sharing behavior. The team can strive to create a good level of trust and create a good organizational atmosphere. On the one hand, the team can train employees and establish a harmonious co-ordination between employees and organizational values. On the other hand, team leaders can constantly refine and innovate organizational culture, improve organizational atmosphere and improve employee satisfaction.

Thirdly, supportive culture moderates the influence of trust tendency on explicit and implicit knowledge sharing behavior. That is, bureaucratic culture moderates the influence of trust tendency on implicit knowledge sharing behavior. Organizations should appropriately build supportive culture and bureaucratic culture. The two cultures have two sides. Specifically, under the low level of supportive culture, the higher the level of trust tendency, the higher the level of explicit and implicit knowledge sharing behavior. Under the high level of bureaucratic culture, the higher the level of trust, the higher the level of implicit knowledge sharing. Under the background of Chinese traditional culture, the team expects the employees to show implicit knowledge sharing. On the one hand, it can build a low-level supporting culture; on the other hand, it can build a high-level bureaucratic culture. Under the two organizational cultures of supportive culture and bureaucratic culture, the higher the level of trust, the more likely the team employees are to perform knowledge sharing.

### Limitations and Directions for Future Research

Like most other empirical studies, this study is also subject to certain limitations. First of all, this study focuses on the differences in the influence of different leadership styles on the knowledge sharing type. Other factors are not considered in this study. Previous research has shown that the type of technology used by organizations can affect trust formation and knowledge Shared by team members. Secondly, the study of leadership style and knowledge sharing behavior mainly relies on cross-sectional data. Future research can be carried out through multi-channel data collection and diversified tools for cross-level analysis. The specific methods include collecting longitudinal data, measuring colleagues and superiors at the same time, and applying tools such as Mplus program for research and analysis. Finally, this study is based solely on Chinese background data collection, which may limit the universality of research results. Future research can be extended to samples from different cultural backgrounds, industries or organizations, such as analyzing the relationship between leadership style and knowledge sharing behavior in different countries and national cultures. It is also one of the future research directions to explore whether cultural differences have differences in the process of leadership influence on knowledge sharing behavior.

## Data Availability Statement

The original contributions presented in the study are included in the article/supplementary material, further inquiries can be directed to the corresponding authors.

## Author Contributions

DJ conceived and designed the experiments and collected and interpretation of the data. ZC analyzed the data, examined and critically contributed to and finally approved the manuscript. All authors contributed to the article and approved the submitted version.

## Conflict of Interest

The authors declare that the research was conducted in the absence of any commercial or financial relationships that could be construed as a potential conflict of interest.

## Publisher’s Note

All claims expressed in this article are solely those of the authors and do not necessarily represent those of their affiliated organizations, or those of the publisher, the editors and the reviewers. Any product that may be evaluated in this article, or claim that may be made by its manufacturer, is not guaranteed or endorsed by the publisher.
